# Effect of Ethanol and Ultrasound Pretreatments on Pineapple Convective Drying

**DOI:** 10.17113/ftb.59.02.21.7045

**Published:** 2021-06

**Authors:** Lívia Dias Campêlo de Freitas, Shirley Clyde Rupert Brandão, João Henrique Fernandes da Silva, Otidene Rossiter Sá da Rocha, Patrícia Moreira Azoubel

**Affiliations:** Federal University of Pernambuco, Department of Chemical Engineering, Av. Prof. Arthur de Sá, s/n, Cidade Universitária, Recife-PE, 50740-521, Brazil

**Keywords:** ascorbic acid, carotenoids, convective drying, product colour, ethanol, ultrasound

## Abstract

**Research background:**

Drying represents a viable unit operation for the preservation of food. Convective drying is the most used method for plant materials. However, it can result in negative changes in food nutrient composition, and other quality parameters, besides requiring high energy consumption. Pretreatments can represent an alternative to minimise these negative aspects of dried materials. This work aims to evaluate the use of ethanol and ultrasound before pineapple convective drying and its effect on the product´s colour, water activity, ascorbic acid and total carotenoid contents.

**Experimental approach:**

For the pretreatment step, fruit samples were immersed in ethanol solutions of different volume fractions, and experiments were carried out for 10 min with and without using ultrasound (25 kHz). Fruit samples were dried at 60 ºC. A control group (without the pretreatment step) was also dried under the same condition. Semi-theoretical models were used for drying data fitting, and the diffusional model was used to describe the moisture transfer and calculate the effective diffusivity. Water activity, ascorbic acid, total carotenoids and colour analyses were performed.

**Results and conclusions:**

The combination of ethanol and ultrasound as a pretreatment reduced the drying time of pineapple. Higher effective moisture diffusivities were obtained when ethanol and ultrasound were applied before drying. The two-term exponential model presented the best fit for drying experimental data. The dried samples had a darker colour than the fresh sample. The pretreatment with ethanol resulted in increased retention of the studied bioactive components. The satisfactory results of this study represent an improvement in the drying process.

**Novelty and scientific contribution:**

Ultrasound and ethanol as a pretreatment to convective drying are promising. However, each food matrix has a typical structure and composition. Therefore, the application of the pretreatment in other products or using other conditions is still necessary to deeply understand and explain their effect on the process and the quality of the dried products.

## INTRODUCTION

 Drying represents a viable unit operation for the preservation of food, which after dehydration, can be directly consumed or rehydrated. Among the different methods, convective drying is the most used for plant materials. However, the use of hot air can result in negative aspects, as some materials change their appearance, texture, colour, nutrient composition and other quality parameters when dried, which displeases the consumer (*1*, *2*). Among these, non-enzymatic browning (appearance/colour) and degradation of nutritional components (nutritional composition) that are thermodynamically unstable or thermosensitive, which is the case with ascorbic acid present in abundance in pineapples (*3*), can be considered as some of the main consequences of drying. Also, convective drying has high energy consumption (*4*). To minimise these negative aspects, pretreatments like ultrasound have been used (*2*, *5*, *6*).

 Ultrasound is sound waves that, when propagated through a biological structure, produce rapid intermittent compressions and expansions, resembling a sponge being squeezed (sponge effect). These compression-expansion cycles promote the phenomenon of cavitation, that is, a rapid series of formation, growth and collapse of bubbles of microscopic dimensions in the plant matrix and, thus, create microchannels that increase diffusivity, and consequently can facilitate the removal of water (*4*).

 Fernandes *et al.* (*7*) studied the use of osmotic dehydration and/or ultrasound in distilled water and sucrose solutions before pineapple drying. They verified that using ultrasound resulted in dried fruits with low sugar content and reductions up to 31% in the drying time. In another work, Fernandes *et al.* (*6*) reported changes in the fruit cell structure and the formation of microscopic channels with osmotic dehydration and ultrasound pretreatment, which could be responsible for the higher drying rates. Corrêa *et al.* (*8*), studying the influence of both ultrasound and osmotic dehydration as pretreatment and also the use of ultrasound during pineapple drying, observed that the ultrasonic waves accelerated drying. However, the authors reported that when only osmotic dehydration was used, lower drying rates were obtained due to increased external resistance. In another study on the use of ultrasound before pineapple drying, Rodríguez *et al.* (*9*) performed the experiments using distilled water and the fruit juice as the soaking medium for the pretreatment step. They reported increased rates with ultrasound application and also better preservation of the fruit bioactive compounds.

 As can be seen, studies on ultrasound as a pretreatment to pineapple drying have been widely published. However, these pretreatments mostly included distilled water, and sucrose solutions of different concentrations. As far as our knowledge goes, only one study on the use of ethanol as the immersion medium for the ultrasonic pre-step of pineapple drying has been carried out, but to obtain iron-fortified pineapple chips (*10*). Also, as Rojas *et al.* (*5*) outlined, there is still the need to understand, describe and improve the mechanisms of using ultrasound, which depends also on the used processing conditions. Also, it is important to estimate its influence on food constituents to resolve if it is an advantage to use it.

 To humans, ethanol is not harmful, and its residue in the dried materials has not been reported. Thus, it can be used as a propagation medium instead of water during the ultrasonic step (*11*). Inside the material, ethanol evaporates before the water, its vapour moves towards the surface, and its flow channels remain as pores, which facilitate the water evaporation (*12*).

 Application of ultrasound and ethanol before convective drying has been investigated recently. Zubernik *et al.* (*1*) investigated the influence of ethanol as a soaking medium for ultrasound pretreatment of apple convective drying. The pretreatment was carried out varying the immersion time, and higher reductions in drying time were obtained the longer the samples were immersed in ethanol before applying ultrasound. However, it did not limit the degradation of the polyphenolic compounds. Cunha *et al.* (*13*) reported higher melon drying rates with increased ethanol volume fraction during ultrasound pretreatment, but also degradation of bioactive constituents and colour. In pumpkin convective drying, Rojas *et al.* (*5*) verified shorter processing time and lower energy consumption, better rehydration characteristics, and lower carotenoid loss for samples pretreated with ethanol and ultrasound. Santos *et al.* (*14*), comparing the use of water or ethanol combined with ultrasound, also reported improved rates and rehydration, and preserved carotenoid content of carrots. Ethanol was also used combined with ultrasound as a pretreatment to other drying methods, such as infrared drying (*4*, *11*). However, it has different mechanisms from convective drying.

Ultrasound and ethanol as a pretreatment to convective drying are promising. However, research with different types of fruits and vegetables, and operating conditions, as well as their influence on quality aspects is still necessary and could provide a basis for other studies and its industrial application (*14*). Thus, this work studied the combined use of ethanol (in different volume fractions) and ultrasound before pineapple convective drying and their effect on the product colour, water activity, ascorbic acid and total carotenoid contents.

## MATERIALS AND METHODS

### Raw material

Perola pineapples (*Ananas comosus*) were obtained from a local market (Recife, Brazil). The fruits were selected according to the maturation characteristics corresponding to size, shape and soluble solid content between 12 and 14 °Brix, measured using a refractometer (model LH-T90; Sinotester, Zhejiang, PR China), to obtain as standardised samples as possible. The samples had a firm pulp when subjected to cutting and no injuries of a pathological, mechanical or physiological nature.

**Fig. S1:**
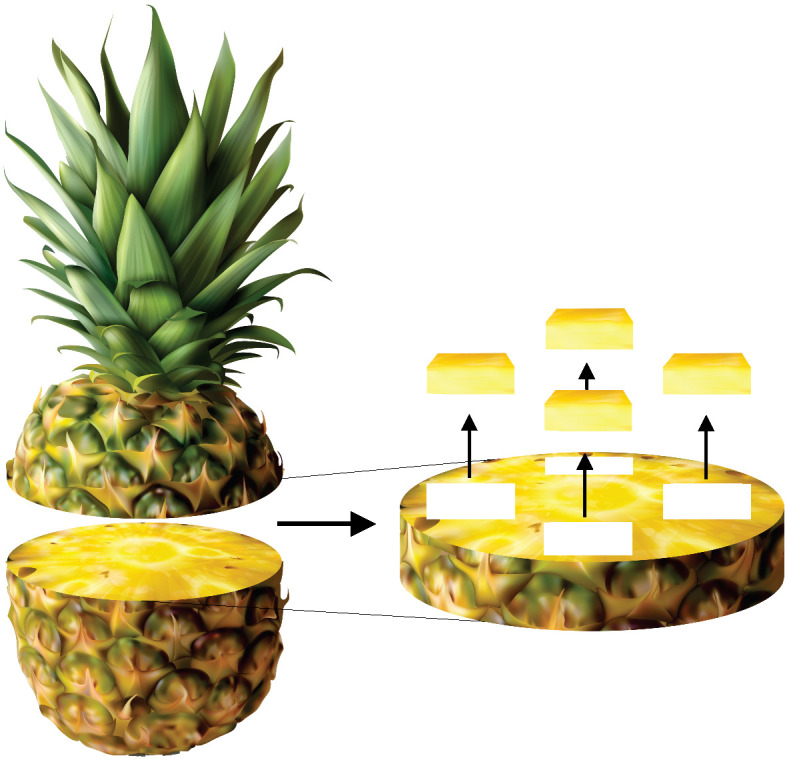
Cutting scheme for obtaining pineapple samples

The pineapples were washed under running water with neutral detergent, dried on absorbent paper and cut into slices. With the aid of a mould and a stainless-steel knife, square samples (2.0 cm×2.0 cm) of 0.2 cm thickness were obtained from the fruit pulp (Fig. S1). The average initial moisture mass fraction on dry mass basis, determined according to AOAC official method 934.06 (*15*), was (5.8±0.2) kg/kg.

### Pretreatment

Absolute ethanol (99.5%, Química Moderna, Barueri, Brazil) was used, as reported by Cunha *et al.* (*13*). The pineapple samples were immersed in different solutions: absolute ethanol (*φ*=100%), without (E100) and with ultrasound treatment (E100US), and aqueous solution with *φ*(ethanol)=50% without (E50) and with ultrasound treatment (E50US).

For all pretreatments, pineapple samples were placed in 250-mL beakers and immersed in ethanol or the aqueous solution with ethanol for 10 min, maintained at 30 °C, using a sample to solution ratio of 1:4 (*m*/*m*). For samples submitted to ultrasound, the beakers were placed in an ultrasonic bath (model USC-2580A; Unique, Indaiatuba, Brazil) with a frequency of 25 kHz (154 W), an intensity of 4870 W/m^2^ (*13*), and temperature control (fluctuations of temperature were avoided by water circulation).

After the pretreatment, the samples were removed from the solution, dried with absorbent paper, weighed and dried using a dryer.

### Drying

The samples with and without (control) pretreatment (approx. 30 g per batch) were dried using a stainless-steel fixed bed dryer (Sulab, Serra Negra, Brazil), air velocity of 2.0 m/s (airflow was perpendicular to the material), and temperature of 60 °C (*13*, *16*-*18*). The sieve load was around 1.5 kg/m^2^. The samples were rapidly removed from the dryer and weighed every 15 min for the first 60 min of drying and, subsequently, every 30 min until reaching constant mass. Experiments were performed in triplicate and the error is less than 0.5%.

For the kinetic study, semi-theoretical models were fitted to the experimental data (Table 1), where *a*, *b*, *c*, *k* and *w* are empirical constants, *t* is the drying time (min), MR is the moisture ratio:


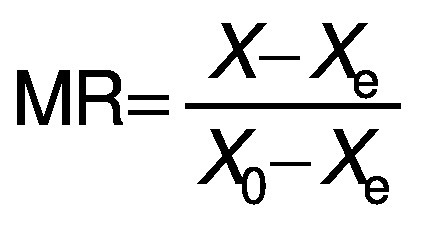


**Table 1:** Thin layer models used for mathematical modelling of pineapple drying

Model	Equation
Single exponential	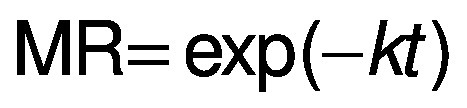
Henderson and Pabis	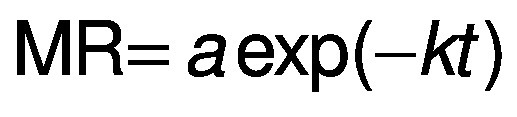
Logarithmic	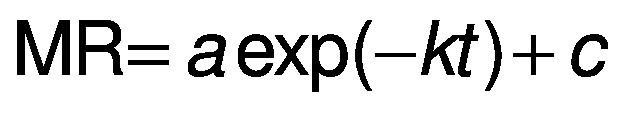
Two-terms	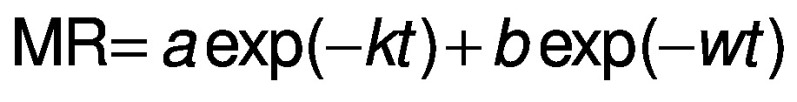
Wang and Singh	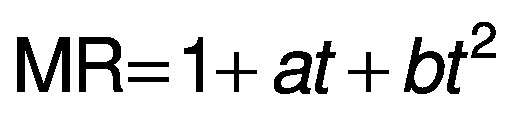

MR=moisture ratio; *a*, *b*, *c*, *k* and *w*=experimental constants; *t*=time

where *X* is the moisture mass fraction on dry mass basis (in kg/kg) at time *t*, *X*_e_ is at the equilibrium and *X*_o_ is the initial moisture mass fraction (in kg/kg).

The model fitting was evaluated by the mean relative deviation module (*E*) and the determination coefficient R^2^:


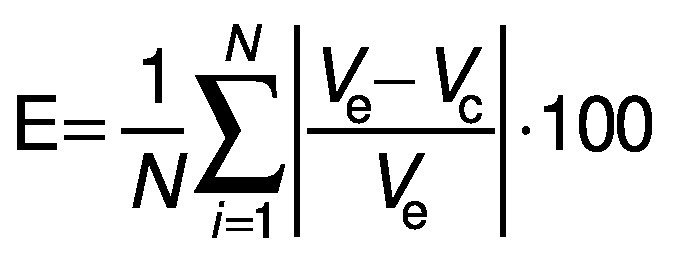


where *N* is the number of experimental data, *V*_e_ is the value obtained using experimental data and *V*_c_ is the calculated value.

The Fick’s diffusion model was used to obtain the effective diffusivity:


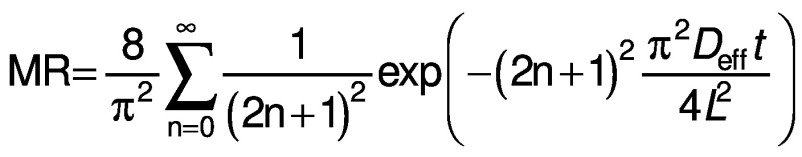


where *D*_eff_ is the effective diffusivity (m^2^/s), *t* is the time (s), *L* is half of the slab thickness (m).

### Quality parameters

For quality assessment, evaluations were performed with fresh and dried pineapple. The latter was dried until reaching a final moisture mass fraction on dry mass basis of 0.19 kg/kg (16% on wet basis), according to the Brazilian legislation for the dried fruit category (*19*). The evaluated quality parameters (in triplicate) were colour, water activity, ascorbic acid and total carotenoid mass fraction.

Ascorbic acid mass fraction was evaluated following Ranganna (*20*), which is based on the reduction of 2,6-dichlorophenol indophenol (Neon, Suzano, Brazil) by ascorbic acid (Química Moderna). The results were expressed on a dry mass basis (mg/100 g).

The total carotenoid mass fraction was quantified based on Rodriguez-Amaya (*21*). In brief, there was an acetone (Química Moderna) extraction, followed by a separation and a dilution in petroleum ether (Química Moderna), finally measuring the absorbance at 470 nm. Some precautions against pigment degradation or alteration were taken, such as protection from light and high temperatures, and the use of a short analysis time. The results were expressed in μg carotenoids per g dry matter.

For colour evaluation, a calibrated colorimeter (model CR400; Minolta, Tokyo, Japan) was used to obtain the parameters: luminosity to darkness (L*=100 to 0), red (+a*) to green (-a*), and yellow (+b*) to blue (-b*). Water activity was measured with a water activity meter (Pawkit, Decagon Devices, Inc, Pullman, WA, USA) at 25 °C.

### Statistical analysis

Experiments were performed in triplicate and the obtained data were submitted to analysis of variance (ANOVA) and Tukey’s test at the 95% confidence level (p<0.05) for comparison between the mean values using the Statistica software v.7.0 (*22*). This software was also used to verify the thin layer model fit to the drying data using the Levenberg-Marquardt non-linear estimation method.

## RESULTS AND DISCUSSION

### Drying kinetics

**Fig. 1:**
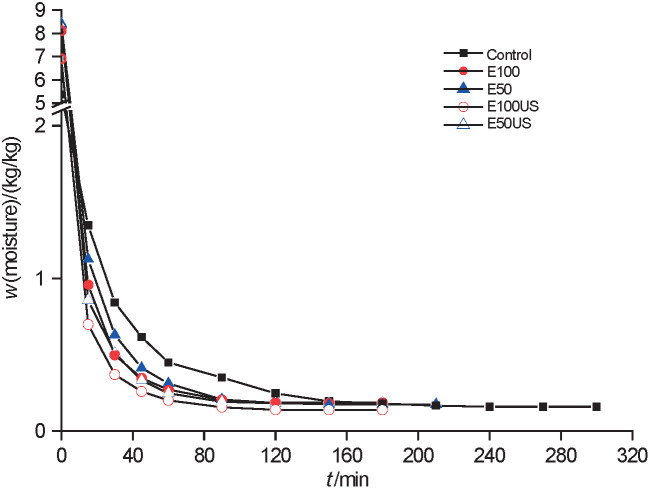
Moisture (*X*) as a function of time (*t*) for the drying process of pineapple with and without pretreatment

**Fig. 2:**
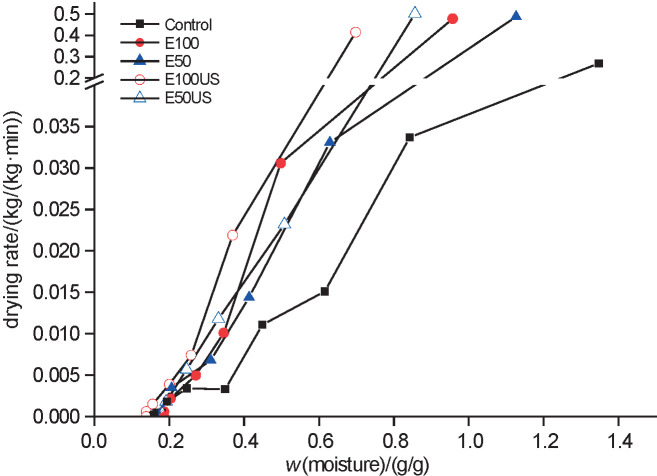
Drying rate as a function of moisture content for the drying of pineapple with and without pretreatment

After the pretreatment, pineapple moisture mass fraction on dry mass basis (in kg/kg) changed to: (8.4±0.4) for E50, (8.1±0.5) for E100, (8.4±0.1) for E50US, and (6.9±0.2) for E100US samples. Fig. 1 and Fig. 2 show the variation in the moisture ratio and drying rates of pineapple, respectively. It can be observed that the pretreated samples had higher drying rates than the untreated ones (control), as indicated by a faster drop in moisture content. The time to reach the dynamic equilibrium (constant mass) of the pineapple samples was 240 min for control (X_e_=0.159 kg/kg), 150 min for E50 (X_e_=0.175 kg/kg), 120 min for the other samples (E100: X_e_=0.186 kg/kg, E50US: X_e_=0.182 kg/kg, and E100US: X_e_=0.139 kg/kg). The pretreatment with ethanol caused a change in the drying kinetics of the pineapple slices, probably due to the Marangoni effect. This is a transfer of mass in the interface of two fluids with different surface tensions, which is favoured by the formed surface tension gradient (*5*). Initially, ethanol, as an organic solvent, dissolves the cell wall compounds, increasing permeability. Afterwards, during drying, it is quickly vapourised. Thus, with the faster vapourisation of ethanol, more water than ethanol remains on the sample surface, generating a region with a higher surface tension that strongly pulls up the water from inside the sample. The process is repeated until a new balance in surface tension is reached (*23*).

The time required to reach a moisture content of 0.19 kg/kg, which is a value within the range allowed by the Brazilian legislation for dried fruits, was 146 min for control, 97 min for E50, 95 min for E100, 85 min for E50US and 60 min for E100US samples. Thus, the use of ethanol together with ultrasound reduced the time of drying to the desired moisture content by more than 70%. Pretreatment with ethanol combined with ultrasound provides marked changes in drying kinetics. The rupture of cell tissue and the formation of microchannels as a result of acoustic cavitation (sponge effect) favour the flow by capillarity and, thus, the Marangoni effect described previously (*4*). Cunha *et al.* (*13*) successfully reduced drying time of melon by 56.9% using ethanol as a pretreatment.

Table 2 shows the parameters of the mathematical models, R^2^ and *E* for drying data fitting. It is observed that the two-term exponential model gave the best fit (R^2^>0.99 and *E*≤10%) and was the one that best represented the experimental data. Medeiros *et al.* (*16*), in the study of the drying kinetics of mango at 60 °C pretreated with ultrasound, also reported that this model obtained the best fit to the experimental data.

**Table 2:** Obtained experimental parameters, coefficients of determination (R^2^) and deviation module (E) of the mathematical models fitted to the pineapple drying kinetic curves

Condition	Parameter	Model				
TT	Log	SE	HP	WS
Control	*a*	0.2516	0.9693	-	0.9919	-0.0164
	*k*	0.0234	0.0946	0.0855	0.0849	-
	*b*	0.7484	-	-	-	0.0001
	*w*	0.1793	-	-	-	-
	*c*	-	0.0263	-	-	-
	R^2^	0.9999	0.9945	0.9919	0.9922	0.3422
	E/%	7.97	271.60	66.04	65.99	1070.06
E50	*a*	0.8075	0.9854	-	0.9993	-0.0263
	*k*	0.0440	0.1474	0.1377	0.1377	-
	*b*	0.1925	-	-	-	0.0001
	*w*	0.0419	-	-	-	-
	*c*	-	0.0143	-	-	-
	R^2^	0.9999	0.9991	0.9984	0.9984	0.6483
	E/%	7.57	228.09	58.99	59.00	3129.75
E100	*a*	0.1455	0.9878	-	0.9997	-0.0317
	*k*	0.0501	0.1606	0.1510	0.1510	-
	*b*	0.8545	-	-	-	0.0002
	*w*	0.2438	-	-	-	-
	*c*	-	0.0121	-	-	-
	R^2^	0.9999	0.9994	0.9922	0.9992	0.7532
	E/%	3.82	76.61	53.24	53.24	1650.16
E50US	*a*	0.1752	0.9888	-	0.9997	-0.0318
	*k*	0.2606	0.1725	0.1617	0.1616	-
	*b*	0.8248	-	-	-	0.0002
	*w*	1.7108	-	-	-	-
	*c*	-	0.0116	-	-	-
	R^2^	0.9999	0.9997	0.9991	0.9991	0.7444
	E/%	9.50	137.32	54.73	54.74	2758.27
E100US	*a*	0.8748	0.9890	-	0.9998	-0.0318
	*k*	0.2774	0.1725	0.1623	0.1623	-
	*b*	0.1252	-	-	-	0.0002
	*w*	0.0437	-	-	-	-
	*c*	-	0.0109	-	-	-
	R^2^	0.9999	0.9996	0.9993	0.9993	0.7423
	E/%	0.44	64.07	54.15	54.16	1622.49

TT=two terms, Log=logarithmic, SE=single exponential, HP=Henderson and Pabis, WS=Wang and Singh, US=treated with ultrasound, E50 and E100=samples pretreated with *φ*(ethanol)=50 and 100%, respectively

The effective diffusivity (*D*_eff_) values for the dried pineapple samples with and without pretreatments are observable in Table 3. All pretreated samples obtained higher values of *D*_eff_ than the control dried pineapple. The use of ethanol (Marangoni effect) with ultrasound (formation of microchannels by cavitation) supported the migration of water from the interior of the sample to the surface, increasing the diffusivity from 2.30∙10^-9^ m^2^/s (control) to 6.23∙10^-9^ m^2^/s (E100US). This represents an increase of 171%. Rojas and Augusto (*4*) observed similar behaviour in the infrared drying of potato slices pretreated with ethanol and ultrasound.

**Table 3:** Diffusivity (*D*_eff_) values for pineapple drying

Treatment	*D*_eff_ ∙10^9^/(m^2^/s)	R^2^	
Control	2.30±0.03		0.9938	
E50	5.18±0.02		0.9984	
E100	5.75±0.01		0.9991	
E50US	6.20±0.01		0.9990	
E100US	6.23±0.02		0.9992	

US=ultrasound treated; E50 and E100=samples pretreated with *φ*(ethanol)=50 and 100%, respectively

### Water activity, total carotenoid and ascorbic acid content, and colour evaluation

Table 4 shows the results of pineapple quality analysis. Pineapple drying significantly reduced the water activity (*a*_w_), as expected. Although significant differences (95% confidence level) were not observed between the dried samples with and without pretreatment, pineapple immersed in higher ethanol volume fraction obtained the lowest *a*_w_ values, which contributes to higher microbiological stability of the dried fruit. Zubernik *et al.* (*1*) reported that there was a significant influence of the pretreatments on hygroscopic properties and the structure of dried apples, and that the ethanol immersion, irrespectively of the application of ultrasound, resulted in decreased water vapour adsorption abilities, causing better stability during storage.

**Table 4:** Physicochemical characterisation of fresh and dried pineapple samples

Sample	*a*_w_	*w*(total carotenoids)/ (μg/g)	*w*(ascorbic acid)/(mg/100 g)	Colour L* a* b*		
Fresh	(0.98±0.05)ª	(5.1±0.1)^a^	(65.26±0.11)^a^	(61.5±0.8)^a^	(-1.33±0.02)^a^	(19.2±0.8)^a^
Control	(0.65±0.01)^b^	(1.23±0.2)^b^	(58.84±0.89)^b^	(52.4±1.8)^b^	(3.4±1.8)^b^	(25.9±2.0)^b^
E50	(0.65±0.02)^b^	(2.1±0.2)^c^	(61.1±0.9)^bc^	(51.8±1.1)^b^	(0.5±0.1)^c^	(22.64±0.02)^c^
E100	(0.61±0.01)^b^	(2.0±0.2)^c^	(63.6±1.0)^c^	(55.9±1.6)^c^	(-0.4±0.14^d^	(22.8±1.1)^c^
E50US	(0.62±0.02)^b^	(1.3±0.2)^b^	(64.2±0.9)^bc^	(57.7±0.8)^cd^	(-0.5±0.2)^d^	(24.8±0.5)^b^
E100US	(0.61±0.02)^b^	(1.4±0.3)^b^	(63.88±0.01)^c^	(59.2±2.0)^ad^	(-0.78±0.07)^e^	(385±0.6)^d^

Samples with the same letter within the same column showed no statistically significant difference for their mean values at 95% confidence level.

US=ultrasound treated; E50 and E100=samples pretreated with *φ*(ethanol)=50 and 100%, respectively; *a*_w_=water activity

Carotenoids are compounds sensitive to factors such as heat and processing time, as these pigments are highly unstable and susceptible to degradation or isomerisation (*24*). Thus, there were significant losses for all dried pineapples compared to the fresh fruit. The dried untreated sample (control), which required the longest drying time, had the lowest total carotenoid mass fraction (Table 4), showing a 76% reduction compared to the fresh sample. Silva Júnior *et al.* (*17*) also reported higher losses for dried untreated papaya, since this sample required a longer processing time.

Carotenoid values without a statistical difference (95% confidence level) from that of control dried samples were found in the samples treated with ultrasound (E50US and E100US). Although these samples required the shortest drying time, the structural changes in the sample matrix caused by ultrasound (rupture of the cell wall and formation of microchannels) seemed to have increased the extraction (or loss) of carotenoids, as observed by Medeiros *et al.* (*16*) in mango drying with and without sound waves.

Among the pretreated samples, the pineapple pretreated in ethanol without ultrasound had lower carotenoid loss. Carotenoids are mostly soluble in organic solvents, such as ethanol, and they can migrate to the pretreatment solution, thereby decreasing their quantity after drying (*5*, *12*). However, Rojas *et al.* (*5*) reported that the effect of pretreatment on the extraction of carotenoids was negligible, due to the small contact area of the pumpkin samples, contrary to when ethanol and/or ultrasound are used for solid-liquid extraction.

 Pineapple drying resulted in significantly (95% confidence level) lower ascorbic acid mass fraction (Table 4). Heat (thermal degradation) strongly affects the loss of ascorbic acid. However, the retention of this compound was equal to or greater than 90% after drying. Previous studies reported higher losses during drying of fruit and vegetables (*17*, *25*). In our study, we observed that by applying ethanol pretreatment, we obtained higher retentions than in the dried control (untreated) pineapple. Wang *et al.* (*26*) reported in the drying of scallion that the absorbed ethanol had a protective effect by minimising the contact of ascorbic acid with water, reducing its oxidation and solubility in water. As the pineapple samples not pretreated with ethanol required the longest drying time, thus were longer exposed to heat and oxygen, this also contributed to its higher loss.

 The use of ultrasound in the pretreatment also resulted in an ascorbic acid loss. Silva Júnior *et al.* (*17*) reported the degradation of ascorbic acid in dried papaya pretreated with ultrasound, attributing this to the possible production of hydroxyl radicals by cavitation. However, dried pineapples pretreated using the combination of ultrasound and ethanol had higher retentions, as those samples required shorter drying time. Also, ultrasound possibly resulted in greater ethanol absorption by the sample, thus protecting it against ascorbic acid oxidation.

 Colour is an important parameter for drying quality evaluation. The colour parameters (L*, a* and b*) of fresh and dried pineapples are in Table 4. A significant decrease (95% confidence level) in the lightness is observable after drying. A similar trend was reported for other fruits and vegetables when dried, like papaya (*17*), nectarine (*18*) and apricot (*27*). The darkening of pineapple was higher in control and E50 samples, which may be related to their longer drying time. Also, darkening could be attributed to the degradation of thermosensitive compounds when exposed to heat, oxidation of ascorbic acid and carotenoid degradation, among others. Pretreatment of samples with ethanol and ultrasound resulted in shorter drying time and lower ascorbic acid loss, which could also reduce darkening.

 The parameters a* and b* significantly increased (95% confidence level) for all dried pineapples, indicating an increase in the red and yellow colours. However, a decrease in L* with an increase in a* indicates browning during drying, as observed by Yao *et al.* (*28*) for mango and by Sakooei-Vayghan *et al.* (*27*) for apricot.

## CONCLUSIONS

 The results obtained in this study suggest that the use of ethanol as a pretreatment has a positive influence on the drying of pineapple, with a reduction in drying time, compared to control. The two-term exponential model was the most adequate to describe the drying kinetic data of pineapple with and without ethanol pretreatment. Quality parameters of all dried samples were reduced. Drying resulted in darker samples with increased yellow and red colours. However, compared to the dried untreated pineapple, the use of ethanol pretreatment also resulted in higher effective water diffusivity and drying rate, and improved the ascorbic acid and total carotenoid retention in the dried fruit.
